# Canine serological survey and dog culling ant its relationship with human visceral leishmaniasis in an endemic urban area

**DOI:** 10.1186/s12879-020-05125-0

**Published:** 2020-06-05

**Authors:** Patricia Marques Moralejo Bermudi, Danielle Nunes Carneiro Castro Costa, Caris Maroni Nunes, Jose Eduardo Tolezano, Roberto Mitsuyoshi Hiramoto, Lilian Aparecida Colebrusco Rodas, Rafael Silva Cipriano, Marta Blangiardo, Francisco Chiaravalloti-Neto

**Affiliations:** 1 Department of Epidemiology, School of Public Health, Universidade de São Paulo (USP), Avenida Doutor Arnaldo 715, São Paulo, SP 01246-904 Brazil; 2 Department of Animal Health and Production, School of Veterinary Medicine, Unesp, Araçatuba, SP Brazil; 3 Center for Parasitology and Mycology, Instituto Adolfo Lutz, São Paulo, SP Brazil; 4 Regional Service 9, Superintendência de Controle de Endemias, Araçatuba, SP Brazil; 5 Zoonosis Control Center, Araçatuba, SP Brazil; 6 MRC-PHE Centre for Environment and Health, Department of Epidemiology and Biostatistics, Imperial College, Norfolk Place, London, UK

**Keywords:** Visceral leishmaniasis, Ecological study, Control measures, Brazil

## Abstract

**Background:**

Visceral leishmaniasis is an important but neglected disease that is spreading and is highly lethal when left untreated. This study sought to measure the *Leishmania infantum* seroprevalence in dogs, the coverage of its control activities (identification of the canine reservoir by serological survey, dog culling and insecticide spraying) and to evaluate its relationship with the occurrence of the disease in humans in the municipalities of Araçatuba and Birigui, state of São Paulo, Brazil.

**Methods:**

Information from 2006 to 2015 was georeferenced for each municipality and modeling was performed for the two municipalities together. To do this, latent Gaussian Bayesian models with the incorporation of a spatio-temporal structure and Poisson distribution were used. The Besag-York-Mollie models were applied for random spatial effects, as also were autoregressive models of order 1 for random temporal effects. The modeling was performed using the INLA (Integrated Nested Laplace Approximations) deterministic approach, considering both the numbers of cases as well as the coverage paired year by year and lagged at one and two years.

**Results:**

Control activity coverage was observed to be generally low. The behavior of the temporal tendency in the human disease presented distinct patterns in the two municipalities, however, in both the tendency was to decline. The canine serological survey presented as a protective factor only in the two-year lag model.

**Conclusions:**

The canine serological coverage, even at low intensity, carried out jointly with the culling of the positive dogs, suggested a decreasing effect on the occurrence of the disease in humans, whose effects would be seen two years after it was carried out.

## Background

Visceral leishmaniasis (VL) is considered a neglected tropical disease. It presents a high fatality rate, which can attain 100% if left untreated [1, 2]. In Brazil, VL control is based on the Visceral Leishmaniasis Control Program (VLCP), the main strategies of which include the diagnosis and timely treatment of human cases, environmental management and chemical control of the vector with residual insecticide spraying, and the canine serological survey and the canine culling of positive dogs in order to reduce the vectors’ sources of infection, since the domestic dog is considered the main reservoir of the disease in urban areas [3].

Despite decades of investment in these control measures in the country, in recent years we have observed the expansion of the transmission areas combined with the emergence of autochthonous cases in previously disease-free areas [4]. In addition, there are also doubts regarding the effectiveness of control measures for disease reduction [5–7]. Furthermore, few studies have been carried out with the objective of evaluating the effectiveness of the control measures. Those few studies have methodological problems that have been recognized in their experimental designs [8].

In the state of São Paulo, Brazil, the first autochthonous cases of VL in humans (HVL) occurred in 1999 in Araçatuba and Birigui, contiguous municipalities located in the northwestern region of the state, followed by other municipalities in the state. These two municipalities have been classified as an area of intense transmission of VL [3, 9, 10].

Because cases of HVL arise two years after the occurrence of canine cases [11], it was decided to test the hypothesis that the effects of VL control measures on the incidence of HVL can be observed after a period of 1 to 2 years. Thus, the objective of this study was to measure the coverage of two VL control activities and one VL surveillance measure in an endemic urban area and to evaluate their relationship with the occurrence of VL in humans. The VL control methods evaluated are: i) insecticide spraying for vector control; ii) culling of the seropositive dogs; and the iii) the canine serological survey.

For our knowledge, this is the first study to propose a spatio-temporal method for modeling the variability of VL and the effects of control measure covariates. Other studies related to VL used spatio-temporal modeling in a Bayesian approach, however, evaluating other effects, such as demographic, socioeconomic, climatic and environmental [12, 13].

## Methods

### Study area, type and period

**Fig. 1:**
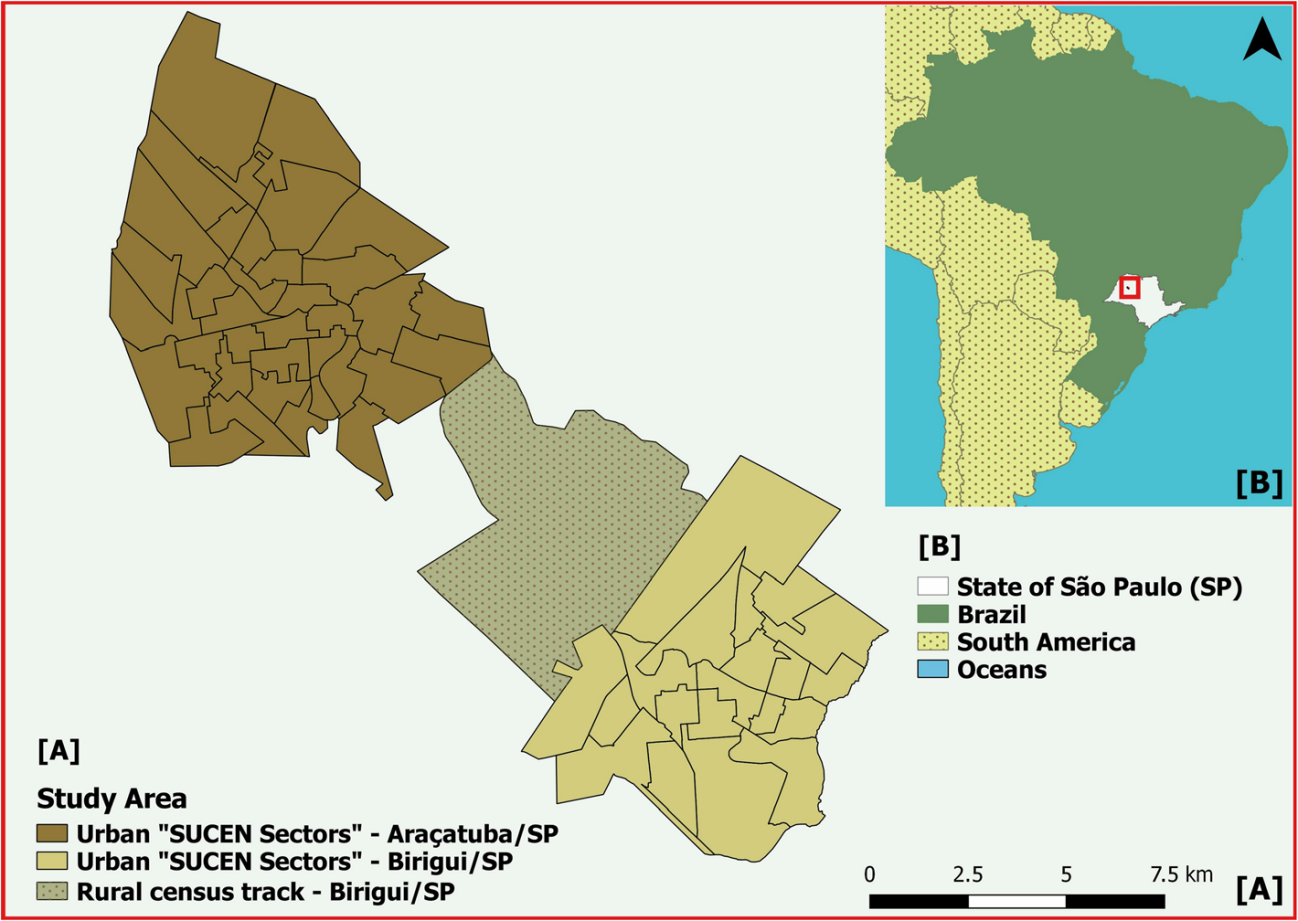
Map of the study area composed of the municipalities of Birigui and Araçatuba. **a** Urban areas of the municipalities of Araçatuba and Birigui and rural area of the municipality of Birigui, State of São Paulo, Brazil. **b** Indication of the area of study, represented by the red rectangle, located in the State of São Paulo, Brazil, and South America. Maps were created by the authors with shape files from: https://www.ibge.gov.br/geociencias-novoportal/downloads-geociencias.html

This is an ecological study using data from 2006 to 2015 and the framework we used is the classical one to investigate spatio-temporal variability in an ecological setting [14, 15]. The study area considered was the urban region of the municipalities of Araçatuba (21° 12′ 41″ S; 50° 25′ 34″ W) and Birigui (21° 17′ 19“ S; 50° 20’ 24” W), plus a rural census tract of Birigui, as a connecting area between them (Fig. 1). The study area is located in the northwest of the state of São Paulo. We chose these municipalities as they were the first to present autochthonous HVL in the state of São Paulo and they are adjacent, thus allowing us to consider them as a single study area.

### Sources of information and GEOREFERENCING

To apply zoonosis control measures, the municipalities divide the urban area into sectors (denominated as “SUCEN Sectors”) that were chosen as the units of analysis (Fig. 1). Additionally, the Brazilian Institute of Geography and Statistics (IBGE) provides the population census data every 10 years and they divide the area in a different track (nominated as IBGE census track). Thus, digital maps of the area were constructed referencing both SUCEN sectors and IBGE census track, using the latter for the rural area.

The Zoonoses Control Center of the respective municipalities provided information according to the units of analysis and year of the human cases (residential address and year of occurrence), the control activities (number of dogs with seropositive tests for VL culled and number of properties sprayed with insecticide), and the surveillance measure (number of dogs evaluated in the canine serological survey).

The diagnostic tests used to identify infected dogs are the TRdPP®-Bio-Manguinhos test for screening and the ELISA test as a test to confirm the previous results. The TRdPP®-Bio-Manguinhos advertises to have a sensitivity of 92.9–100% and specificity of 87.5–91.7%, compared to parasitological test. The ELISA test presents sensitivity of 94.54% and specificity of 91.76%, compared to indirect immunofluorescence test [16]. The seropositive dogs are culled according to the laws with ethical principles mentioned in the surveillance manual [9]. The insecticide used in spraying operations is Alfacypermetrina with 40 mg of ingredient dose active per m^2^ and with a loading weight of 50 ml. This weight of the load was calculated for use in a standard spray pump with 10 l of capacity [9].

The human cases were geocoded using their residential address and information on the control measures was joined with the “SUCEN Sectors” map. The populations of the SUCEN sectors were estimated based on the information provided by IBGE (IBGE, 2018) and Foundation for the State System of Data Analysis [17, 18].

The dataset with information of human visceral leishmaniasis incidence, canine seroprevalence and control measures in Araçatuba/SP and Birigui/SP (period 2006–2015), is available according to the official code of municipalities of study in the ‘Additional File 1’ of Supplementary Information.

### Dependent variable

Incidence rates of HVL, according to the year of study, were calculated for Araçatuba and Birigui, dividing each year’s number of cases by the respective populations. After the geocoding of HVL cases, the number of cases of HVL per year, per unit of analysis was obtained.

### Covariates

The following covariates were obtained by unit of analysis and represented the control measure’s coverage:

- Canine serological survey coverage: the ratio between the number of dogs evaluated in the survey, multiplied by 100, and the estimated number of dogs that should be evaluated. The number of dogs was estimated based on information about the human population (ratio of 1 dog: 5 people) [19];
- Canine seroprevalence for *Leishmania infantum*: ratio of number of seropositive dogs, multiplied by 100, and the number of dogs evaluated in a serological canine survey;
- Canine culling coverage: the ratio between the number of dogs that were culled, multiplied by 100, concerning the number of dogs seropositive for VL;
- Insecticide spraying coverage: the ratio between the number of properties that received the chemical control, multiplied by 100, and the number of properties expected to receive this control. This was estimated based on a block of radius of 200 m around each human case.
- We also considered the municipality of occurrence of HVL cases (Araçatuba or Birigui) as a covariate.

### Data analysis

To evaluate the spatio-temporal variation of HLV and the role of the covariates, the natural framework of analyses is that of ecological regression, as the data are provided as number of cases for each municipality and year [20]. The number of cases of HVL was assumed to follow a Poisson distribution, characterized by an offset given by the population in that area and by a rate specified as a latent Gaussian model with the incorporation of a spatio-temporal structure.

Initially, the number of HVL cases was considered to be a function of an intercept function and random spatial and temporal effects (intercept model). As for random spatial effects, they were considered a component of the Besag-York-Mollié (BYM) type, with random effects representing the spatial dependence of the number of cases of HVL as a function of the geographical location of the units of analysis. In particular, the BYM model is the sum of two random effects, one assuming that neighboring areas are correlated (local dependency) and one which assumes that all the areas have a degree of correlation (global dependency) [21]. To represent the temporal autocorrelation present in the data, an autoregressive model of order 1 of the random walk-1 type (RW1) was adopted [22]. This modeling was performed for the entire study period (2006 to 2015).

For the initial construction of covariate models, an exploratory data analysis was performed. Insecticide spraying coverage, according to the year and the unit of analysis, had a large number of values equal to or approximately zero. The canine culling coverage revealed collinearity with the canine serological survey coverage (correlation coefficient of 0.58) and, for this reason, insecticide spraying was not included in the model. The canine seroprevalence for *Leishmania infantum* was, moreover, not included because it was a result of the canine serological survey and not a control measure. Therefore, only the municipality and the canine serological survey coverage were considered in the covariate models.

Three models were tested, the first considering the yearly number of human cases and the canine serological survey coverage of the same year (with no lag) and the second and third models with a respective one and two-year lag (lagged models of 1 or 2 years). The first model considered the number of cases occurring in the period from 2006 to 2015, the second from 2007 to 2015, and the third from 2008 to 2015.

To obtain the number of expected cases, the global incidence rate was calculated by dividing the total number of HVL cases throughout the study period by the sum of the populations of the two municipalities of every year. With this global rate, the number of expected cases for each unit of analysis in each year was obtained by relating the expected rate to the population of the unit of analysis for the respective year. For the intercept model and the covariate model with no lag, the global incidence rate was calculated based on cases occurring between 2006 and 2015. For covariate and lagged models, the respective rates were calculated for the periods from 2007 to 2015 and from 2008 to 2015.

By considering the number of cases expected in the modeling, per year and unit of analysis, we can interpret the coefficients used, after exponentiation, as relative risks (RR) concerning the average incidence rate of the entire study period. The coverage of control activities used in the models were standardized by the respective means and standard deviation.

Modeling was performed in a Bayesian context using the INLA (Integrated Nested Laplace Approximation) methodology, which obtains deterministically the posterior distributions of the parameters of interest. This approach, concerning the computational time consumption, is more efficient than the MCMC (Markov Chain Monte Carlo) method, especially when dealing with latent Gaussian Bayesian models [23].

**Fig. 2:**
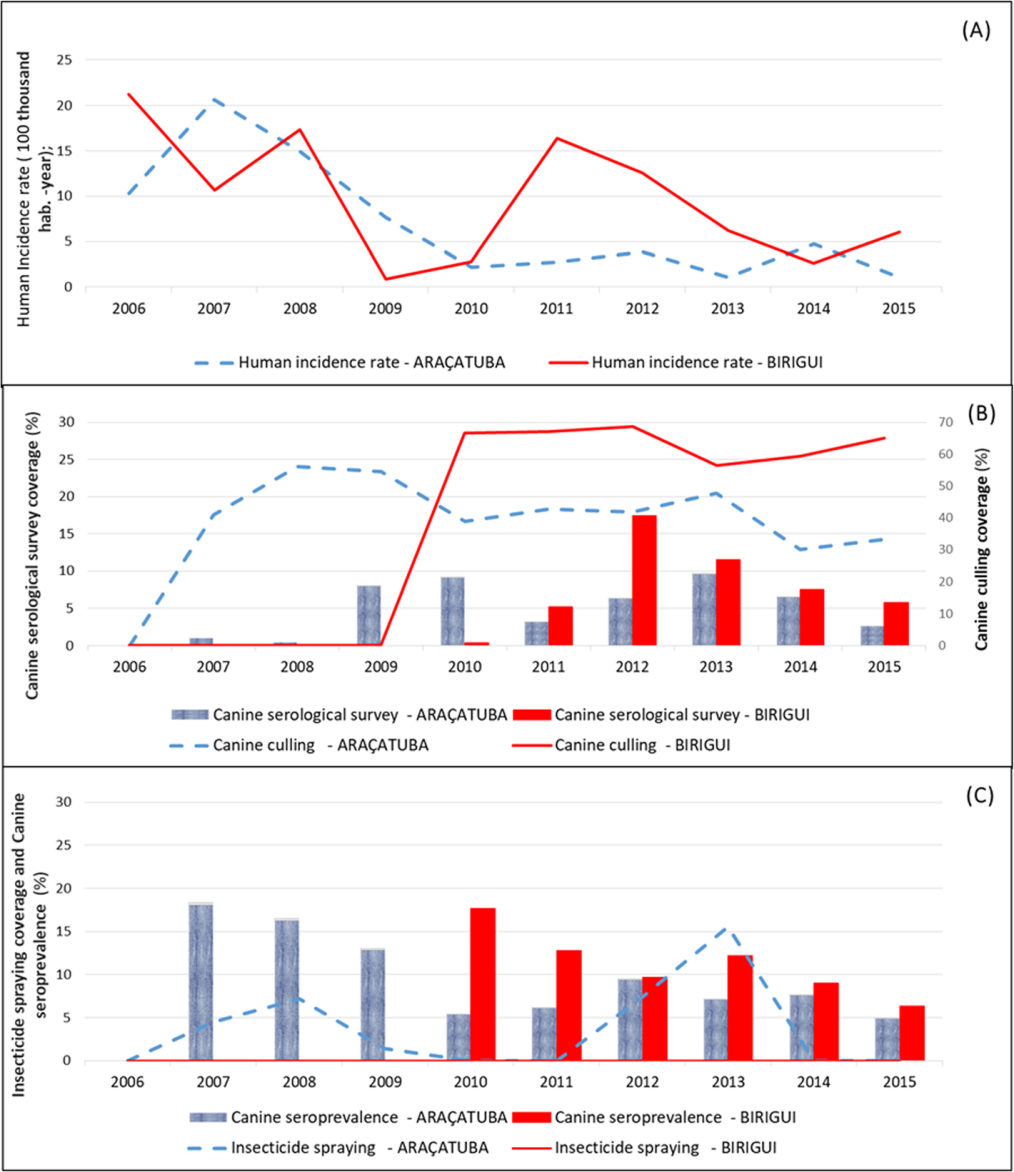
Human Visceral Leishmaniasis incidence rates and control measures, Araçatuba and Birigui, from 2006 to 2015. **a** Incidence rates of human visceral leishmaniasis according to the municipalities of Bririgui and Araçatuba, state of São Paulo, Brazil. **b** Canine serological survey coverage and canine culling coverage in both municipalities. **c** Insecticide spraying coverage and canine seroprevalence according to the municipality. Graphics were created by the authors

**Fig. 3:**
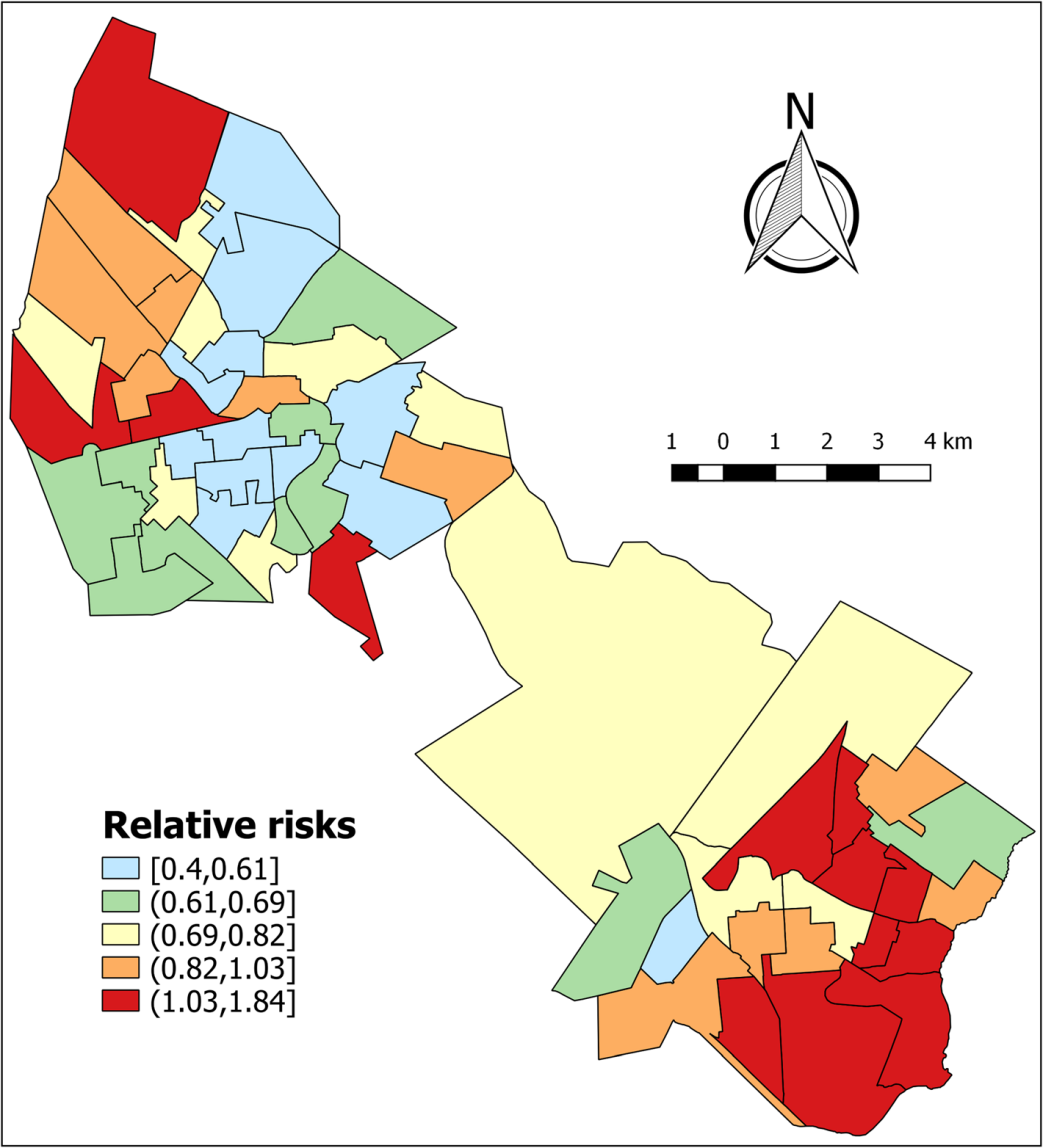
Relative risk posterior means for the human visceral leishmaniasis occurrence for the intercept model. Maps were created by the authors with shape files from: https://www.ibge.gov.br/geociencias-novoportal/downloads-geociencias.html

**Fig. 4:**
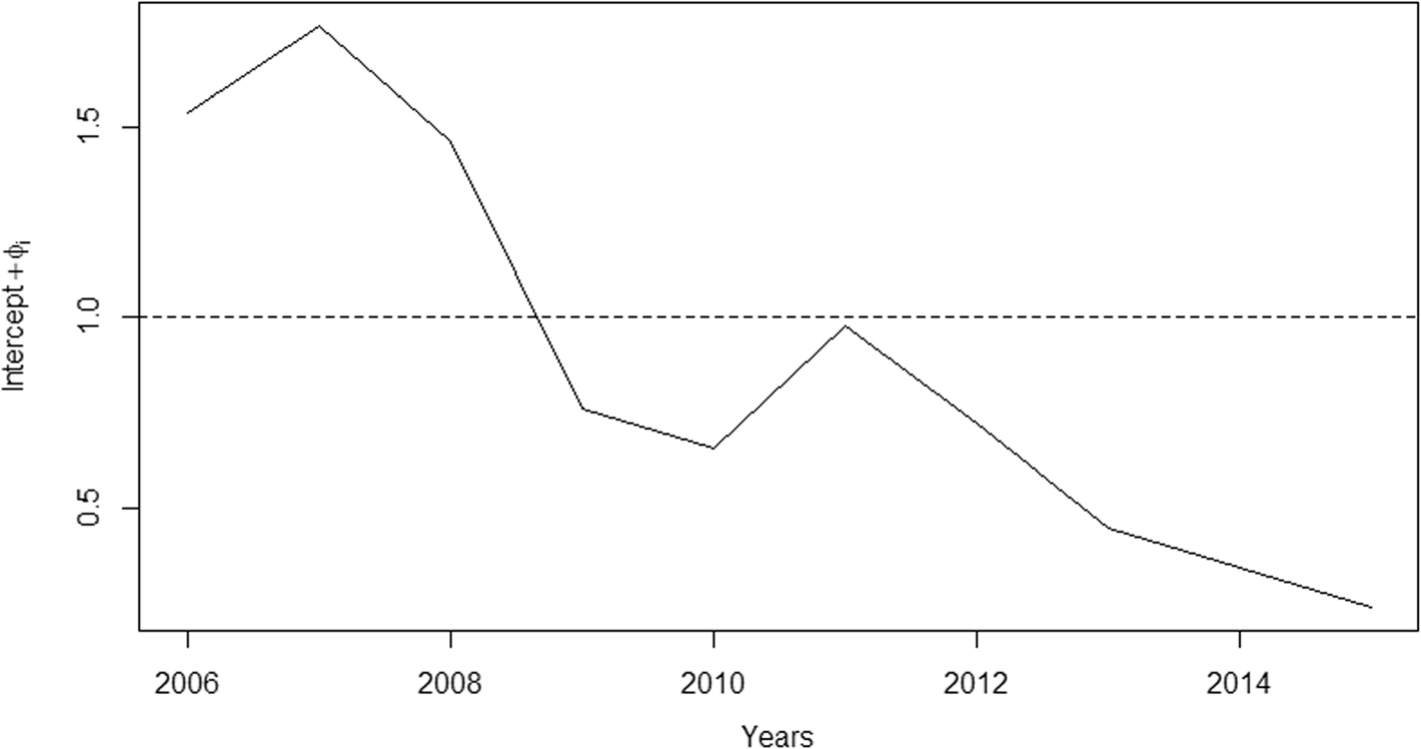
Annual relative risk posterior means for the human visceral leishmaniasis occurrence for the intercept model. Graphic was created by the authors

We used the TerraView software [24] to geocode the LV case address. The software QGIS [25] was used for spatial analysis tools and R software [26] for statistical analysis. The Figs. 1 and 3 were created by the authors, through QGIS Software Development Team (QGIS 2.18.20), available at <http://qgis.osgeo.org> and using the layers obtained from the Brazilian Institute of Geography and Statistics (IBGE), available at <https://www.ibge.gov.br/geociencias-novoportal/downloads-geociencias.html>. The Fig. 2 was also created by the authors using Microsoft Excel, available at <https://office.microsoft.com/excel> and Fig. 4 was created by the authors using R software, available at <https://cran.r-project.org/bin/windows/base/>.

## Results

The HVL incidence in Araçatuba initially decreased and then stabilized in 2010 (Fig. 2a), while in Birigui, incidence continually decreased until 2009, followed by an increase in 2011, and then a subsequent decrease again. The decrease and stabilization of the incidence rates observed in Araçatuba were accompanied by canine serological survey coverage ranging from 0 to 10% (Fig. 2b). In Birigui, the increase in incidence in 2010 and 2011 was preceded by a period in which control activities were suspended (2006 to 2009).

Considering only the years in which canine serological surveys were performed, the HVL incidence curve in Araçatuba (2007 and 2015) behaves similarly to that of canine seroprevalence (Fig. 2c). In Birigui, the decrease in the incidence of HVL (2010 to 2015) presents a one year lag in canine seroprevalence. With regards to the coverage of the control measures, it was observed that the coverage of canine culling was highest, reaching values up to 70% in years in which canine serological surveys were performed (Fig. 2b). On the other hand, insecticide spraying coverage for vector control was very low in Araçatuba, the highest percentage being 15% in 2013 and 0% in Birigui (Fig. 2c). In general, control measures were performed with low coverage, though with greater intensity and consistency in Araçatuba.

The relative risks (RR) for HVL in the SUCEN sectors for the intercept model, from 2006 to 2015, revealed higher values in Birigui than in Araçatuba (Fig. 3). While 60% (12/20) of Birigui’s SUCEN sectors (excluding the rural census track) had RR between the third quintile and the maximum value of RR, in Araçatuba the percentage of sectors lying between these two values was 27.8% (10/36). The annual values of the RR for HVL for the intercept model (Fig. 4) revealed a decreasing temporal tendency, with a peak in 2011.

Figure 3 shows the risk relative for the intercept model (intercept and spatial and temporal random effects) for the human visceral leishmaniasis occurrence in municipalities of Araçatuba and Birigui, state of São Paulo, Brazil, from 2006 to 2015. The graphic presented in Fig. 4 shows the annual relative risk for the intercept model (intercept and spatial and temporal random effects) for the occurrence of human visceral leishmaniasis municipalities of Araçatuba and Birigui, state of São Paulo, Brazil, from 2006 to 2015.

The municipality covariate was considered, in the three covariate models analyzed, significantly and positively associated with HVL incidence. The risk of occurrence of HVL in Birigui was between 1 and 2 times greater in Araçatuba (RR between 1.96 and 2.76). The effect of the canine serological survey coverage and subsequent canine culling of the positive dogs was an important result when they were lagged two years of the incidence of HVL. In this situation, the coverage of the canine serological survey was negatively associated with the occurrence of human cases and seen to have a protective effect on the incidence (RR = 0.76). The increase of one standard deviation in this coverage corresponded to a decrease of 34% in the risk of occurrence of HVL, Table 1.

**Table 1:** Relative risk posterior means for the Human Visceral Leishmaniasis occurrence for three covariate models

Covariate		Delay between HVL occurrence and control measures								
		No lag			One-year lag			Two-year lag		
		(2006 to 2015)			(2007 to 2015)			(2008 to 2015)		
		RR	Limits CI 95%		RR	Limits CI 95%		RR	Limits CI 95%	
			lower	higher		lower	higher		lower	higher
Intercept		0.56	0.41	0.72	0.56	0.41	0.73	0.49	0.32	0.68
Municipality	Araçatuba	1			1			1		
	Birigui	1.98	1.32	2.93	1.96	1.29	2.94	2.76	1.62	4.72
CSSC		1.07	0.92	1.23	1.11	0.94	1.29	0.76	0.55	0.98

The table shows the relative risks and their respective 95% confidence intervals (CI 95%) for three covariate models (intercept, covariates and spatial and temporal random effects) for human visceral leishmaniasis incidence. The first one considered no delay between the disease incidence and the control measures. The second and third models considered a delay between the disease incidence and the control measures, respectively, of one and two years. These results are related to the municipalities of Araçatuba and Birigui, state of São Paulo, Brazil, from 2006 to 2015. CSSC, Canine Serological Survey Coverage.

## Discussion

The finding of this study goes in the same direction as the theoretical study of Costa et al. [27]. These authors suggested, using mathematical models, possible impact on canine VL seroprevalence after the long-term continuous program, targeting both asymptomatic and symptomatic dogs, for lower and moderate transmission of HVL rate scenarios. However, it is worth mentioning that this study did not evaluated the impacts on LVH. In our study, areas are classified as having intense transmission, the intervention coverage was low and, even that, the finding suggested a decreasing effect on the occurrence of the disease in humans, after two years of canine serological coverage, jointly with the culling of asymptomatic and symptomatic positives dogs.

It is worth noting that, in the descriptive part of this study, the result was pointed out that Araçatuba has a peak in the incidence of LVH in 2007, followed by a valley in 2010. After this period, the incidence presented a stabilization. In Birigui, there were two peaks, one in 2006 (about 21 cases / 100 thousand inhabitants-year) and the other in 2011(about 16 cases / 100 thousand inhabitants-year), followed also by stabilization. According to data from the Notifiable Diseases Information System [28] this stabilization continues until 2019. Thus, the incidence of human VL remains below 8 cases / 100 thousand inhabitants per year for at least ten years in Araçatuba and, at least, six years in Birigui.

In Araçatuba and Birigui, due to its territorial characteristics and the intensity of HVL transmission, the visceral leishmaniasis control program (VLCP) recommends that census surveys be carried out to identify naturally infected dogs [3]. It was, therefore, expected that the coverage of the canine serological survey would be 100%. However, it remained below 10% throughout the entire study period. The canine serological surveys only covered some of the sectors of these two municipalities, which was the reason these activities were not carried out as had been expected. This situation occurs in other municipalities elsewhere in the country and can be explained by public resistance to the VLCP, by structural difficulties from municipal administration [29] or by underfunding and lack of political support for the VLCP [8].

Results indicate that even in more populous municipalities, control measures targeting the canine reservoir in high priority areas may be effective at reducing human incidence. The effectiveness of the canine serological survey in reducing the risk for HVL, observed in this study (less than 40%), suggests that increasing the coverage of the serological survey, to the percentage recommended by the VLCP and may result in an even greater risk reduction. However, it is noteworthy that, as pointed out by other authors, given the operational challenges for the complete implementation of the control activities recommended in the VLCP, there are doubts about the feasibility of the suggested coverage levels [8, 29–31].

The coverage of positives dog culling, which is dependent on the canine serological survey, was high where the latter activity was performed. It is already known that when the infected dogs are eliminated the source of infection for the vector in that locality is reduced [32, 33]. Moreover, some studies suggest that canine culling has a lower cost compared to vector control [34, 35].

Some authors have found results similar to those of this study regarding the effectiveness of canine control. Costa et al. [36] demonstrated that canine culling reduced the incidence of HVL by 80% when compared to areas that only received Insecticide spraying in Teresina, PI, Brazil. Nunes et al. [37], moreover, revealed, in a two-year lag analysis, that the reduction in the incidence of HVL was statistically correlated with the increase in canine culling. Werneck et al. [38] showed that, as compared to insecticide spraying, only canine culling was effective in reducing the incidence of HVL, presenting an inverse relationship between these measures. Although those authors considered the positivity of the result to be biased due to the selective loss during follow-up, our results reinforce their findings.

Nevertheless, there is no consensus in the literature about the effectiveness of canine reservoir elimination. Dietze et al. [39] showed that canine culling did not result in a significant decrease in human incidence, pointing to the possibility of humans being reservoirs. Ashford et al. [40], although they suggested that eliminating the majority of the positive dogs could reduce the human incidence, concluded that the attempt to remove them was insufficient to guarantee the elimination of the disease. Thus, they suggested that other reservoirs could be acting in the transmission, while also questioning the serological tests for the identification of the seropositive dogs. In addition, some authors point to problems related to canine cullings such as the lack of precision of the serological tests, the long delay between the identification and the canine culling, the refusal of the owners to cooperate and the replacement of the dogs that were culled [31, 38, 41, 42].

In addition to the technical issues, the most controversial aspect of canine reservoir elimination has been the ethical question considering the emotional involvement of both the owners and the subjects involved in the culling procedure [33–46]. However, it is noteworthy that HVL is a disease with high lethality if untreated, and it is in the process of geographical expansion [3, 47]. Thus, to stop canine reservoir control measures, it is first necessary that there is an efffective alternativepublic health programs.

Among these alternatives are the canine treatment, canine vaccination and the use of the insecticide-impregnated collar, available so far through the initiative of the owners. To date, there is canine treatment that was legally approved by the Ministries of Agriculture and Health in 2016, highlighting the fact that parasitological cure has not yet been proven. This treatment, in addition to being costly, requires the use of the insecticide-impregnated collar and repellent against the vector, as well as a follow-up of the canine patient by a veterinarian every 4 months, which makes it difficult to apply to the population in general [48, 49]. However, there are published recommendations of preserving the right to choose between responsible treatment and euthanasia for owners of infected dogs [50].

Dog vaccination, whose efficacy ranges from 58.1 to 80.8%, has been considered a good alternative [51], however, there is a lack of randomized studies that might demonstrate its efficacy for general use as a measure of VL prevention [42, 52]. In addition to the cost, one of the difficulties for large-scale dog vaccination is the need for three initial doses, with annual boosters, a strategy that is impractical for public health programs.

The use of insecticide-impregnated collars has been recommended as the most effective measure among those cited above [43, 53–55]. Silva et al. [56] demonstrated a reduction of 15% (*p* = 0.004) in Montes Claros, MG, Brazil, and 60% (*p* < 0.001) in Fortaleza, CE, Brazil, in the vector population of captured. Kazimoto et al. [57] observed that there was a 53–59% reduction in the VL incidence in dogs resulting from the use of these collars. However, this measure requires continuous use in a large percentage of the canine population. Also, the collars must be changed every six months and replaced when lost. As a result, the authors warn of their high cost when used on a large scale [56, 58, 59].

One of the limitations of our study was the fact that insecticide spraying had zero or very low coverage in the municipalities studied, making it impossible to evaluate its effectiveness in reducing HVL incidence. This low coverage is indicative of the operational difficulties faced in the implementation, including the residents’ refusal to apply insecticide in their homes, deficiencies in the quality control of the handling of the insecticide, a brief residual effect of the insecticide spraying and a lack of human and financial resources [7, 36, 60]. Another limitation is the fact that both dogs and humans move and there is no way to be sure that transmission of Leishmania occurred in the SUCEN sector where cases were assigned.

Another issue that must be considered is the lack of accuracy of available serological tests, especially for asymptomatic dogs. Grimaldi et al. [61] highlight the TRdPP®-Bio-Manguinhos presents high sensitivity (98%) for diagnosed dogs with symptoms, but low sensitivity to identify dogs without signs and symptoms (47%). Moreover, these authors discuss that the ELISA test, despite having reliable sensitivity (ranging from 93 to 100%) for symptomatic dogs, also presents less sensitivity for dogs without symptoms. It is noteworthy that, even using tests with low sensitivity for asymptomatic dogs, our results suggested an inverse relationship between HVL incidence and canine survey.

Moreover, we were unable to consider controlling the model for HIV/AIDS incidence during the study period. HIV/ AIDS coinfection is known associated with higher risk of VL human symptomatic infection and has important epidemiological and clinical implications. In endemic areas, it is common for cases of VL to be asymptomatic. However, HIV coinfection increases the risk of developing symptomatic VL by between 100 and 2320 times [62]. Since it was not possible to adjust our models for the incidence of HIV / AIDS, it is not possible to state what effect this variable would have on the relationship between the incidence of HVL and the canine serological survey coverage, an issue that needs to be investigated in new studies.

Another limitation is the use of secondary data. Nevertheless, these data were indispensable and appropriate for the development of the study. It is worth noting that it is the health teams which make the decisions about the planning of the surveillance actions and control of VL in their respective municipalities based on this information. This information is essential for all involved in VL control efforts.

Strengths of the study include the consideration of the spatial and temporal dependence of the phenomenon studied and, consequently, more accurate estimates were obtained than in studies that did not take these dimensions into account. The proximity of Araçatuba to Birigui is also worth mentioning as it made it possible to evaluate the relationship between HVL and control measures more comprehensively and with greater study power, given the greater number of units of analysis.

## Conclusions

Our study revealed that the canine serological survey in the municipalities of Araçatuba and Birigui, undertaken concomitantly with the canine culling of positive dogs, although with incomplete coverage, suggests a reduction in the risk for the occurrence of HVL of 34%, with the effects observed after two years of the implementation of these control measures. Therefore, the study suggest that, even when below the recommended levels, the control measures directed at the canine reservoir were effective for the control of HVL.

Thus, our study showed the significant role played by the domestic dog in the occurrence of human disease. It suggests the use of measures for surveying and eliminating these reservoirs should continue until new control measures are proven effective as a large scale alternative reduce the risk of visceral leishmaniasis in humans. Others studies with different methodological approaches must be carried out to affirm the efficacy of this association.

## Supplementary information

**Additional file 1: Table S1:** Dataset with information of human visceral leishmaniasis incidence and control measures. The dataset contains information on the incidence rates of human visceral leishmaniasis, control measure coverages, and canine seroprevalence for *Leishmania infantum*, according official code of municipalities of study, Araçatuba/SP and Birigui/SP and period 2006–2015. The data sources were the Zoonoses Control Center of Araçatuba/SP and Birigui/SP.

## Data Availability

All relevant data are within the Additional File 1.

## Abbreviatons

CI: Confidence intervals
CSSC: Canine Serological Survey Coverage
HVL: Human Visceral Leishmaniasis
IBGE: Brazilian Institute of Geography and Statistics
BYM: Besag-York-Mollié
INLA: Integrated Nested Laplace Approximation
MCMC: Markov Chain Monte Carlo
RR: Relative risks
RW1: Random walk-1 type
VL: Visceral leishmaniasis
VLCP: Visceral leishmaniasis control program
